# Neurobiology of KB220Z-Glutaminergic-Dopaminergic Optimization Complex [GDOC] as a Liquid Nano: Clinical Activation of Brain in a Highly Functional Clinician Improving Focus, Motivation and Overall Sensory Input Following Chronic Intake

**DOI:** 10.23937/2378-3656/1410104

**Published:** 2016-05-11

**Authors:** Lucien L Duquette, Frank Mattiace, Kenneth Blum, Roger L Waite, Teresa Boland, Thomas McLaughlin, Kristina Dushaj, Marcelo Febo, Rajendra D Badgaiyan

**Affiliations:** 1 New Pathway Counseling Services Inc., Paramus, NJ, USA; 2 Behavior Wellness Center, Englewood, NJ, USA; 3 Department of Psychiatry & McKnight Brain Institute, University of Florida College of Medicine, Gainesville, FL, USA; 4 Division of Addiction Services, Dominion Diagnostics, LLC., North Kingstown, RI, USA; 5 Division of Neuroscience-Based Therapy, Summit Estate Recovery Center, Los Gatos, CA, USA; 6 Department of Psychiatry & Behavioral Sciences, Keck School of Medicine of USC, Los Angeles, CA, USA; 7 Department of Clinical Neurology, PATH Foundation NY, New York, NY, USA; 8 Department of Nutrigenomic Translational Research, LaVita RDS, Salt Lake City, UT, USA; 9 Division of Neuroscience Research & Addiction Therapy, Shores Treatment & Recovery Center, Port Saint Lucie, FL, USA; 10 Total Health Solutions, Inc., Miami Beach, FL, USA; 11 Center for Psychiatric Medicine, North Andover, MA, USA; 12 Department of Psychiatry, Laboratory of Molecular and Functional Imaging, University of Minnesota, Minneapolis, MN, USA

**Keywords:** Glutaminergic, Dopaminergic, Reward Deficiency Syndrome (RDS), KB220z-nano-liquid Variant, Focus, Motivation, Sensory input

## Abstract

**Background:**

With neurogenetic and epigenetic tools utilized in research and neuroimaging, we are unraveling the mysteries of brain function, especially as it relates to Reward Deficiency (RDS). We encourage the development of pharmaceuticals or nutraceuticals that promote a reduction in dopamine resistance and balance brain neurochemistry, leading to dopamine homeostasis. We disclose self-assessment of a highly functional professional under work-related stress following KB220Z use, a liquid (aqua) nano glutaminergic-dopaminergic optimization complex (GDOC).

**Case presentation:**

Subject took GDOC for one month. Subject self-administered GDOC using one-half-ounce twice a day. During first three days, unique brain activation occurred; resembling white noise after 30 minutes and sensation was strong for 45 minutes and then dissipated. He described effect as if his eyesight improved slightly and pointed out that his sense of smell and sleep greatly improved. Subject experienced a calming effect similar to meditation that could be linked to dopamine release. He also reported control of going over the edge after a hard day’s work, which was coupled with a slight increase in energy, increased motivation to work, increased focus and multi-tasking, with clearer purpose of task at hand. Subject felt less inhibited in a social setting and suggested Syndrome that GDOC increased his Behavior Activating System (reward), while having a decrease in the Behavior Inhibition System (caution).

**Conclusion:**

These results and other related studies reveal an improved mood, work-related focus, and sleep. These effects as a subjective feeling of brain activation maybe due to direct or indirect dopaminergic interaction. While this case is encouraging, we must await more research in a larger randomized placebo-controlled study to map the role of GDOC, especially in a nano-sized product, to determine the possible effects on circuit inhibitory control and memory banks and the induction of dopamine homeostasis independent of either hypo- or hyper-dopaminergic traits/states.

## Introduction

In the treatment of Reward Deficiency Syndrome (RDS) [1], there are many potential therapeutic targets emerging, and a brief review of some neurochemical pathways seems parsimonious. These pathways include the serotonergic, cholinergic, endorphinergic, glutaminergic, and dopaminergic. However, based on current knowledge, we are proposing that a gentle induction of “dopamine homeostasis,” instead of blocking dopamine, is tantamount to successful long-term treatment as well as relapse prevention in the heroin or opiate/opioid dependent person [1–4]. According to Li *et al.* [3], compared with non-relapsers, those who do relapse from heroin exhibited considerably higher cue-induced cravings as seen on functional magnetic resonance imaging (fMRI), and this brain reaction occurred mainly in the bilateral nucleus accumbens (NAc)/subcallosal cortex and cerebellum. Moreover, the difference in desire positively correlated with the initiation of cue-induced cravings in the NAc/subcallosal cortex among patients. These results indicate that in heroin-dependent persons seeking treatment, higher cue-induced cravings and particular increased area activations may be linked to reward/craving and memory recovery functions. Most importantly, these responses may predict relapse and represent important targets for the development of new treatment for heroin addiction, possibly via regulation of brain dopaminergic function.

**Figure 1:**
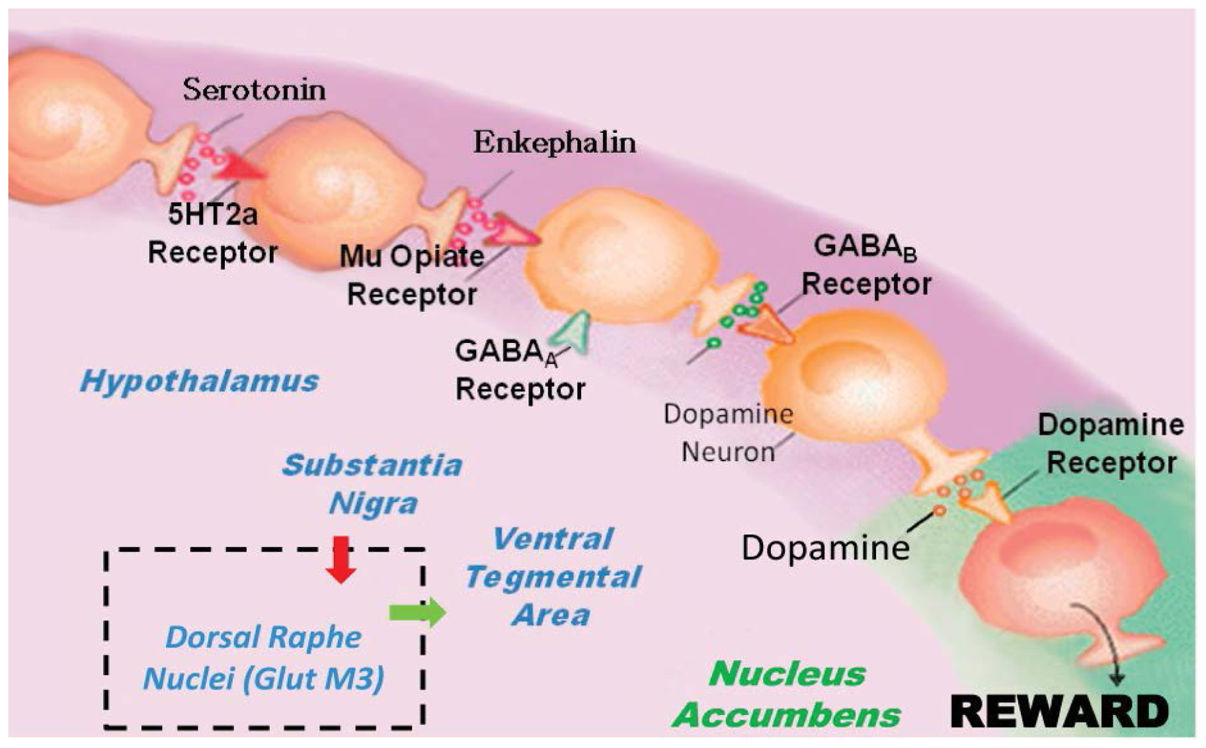
Brain Reward Cascade showing the inclusion of Dorsal Raphe Nuclei (GlutM3) interaction regulating Ventral Tegmental Area (VTA) dopamine. With Permission [1].

To fully appreciate the distinct role of any individual neuropath way, it is important to evoke the concept of “Brain Reward Cascade” first developed by Blum & Kozlowski [5,6] as previously indicated by Bozarth & Wise [7]. In the Bozarth and Wise paper, they correctly suggest that heroin reward is dependent on a dopaminergic substrate, and the cascade intimates the various interactions of a number of neurotransmitters leading to NAc dopamine release. Many reiterations have been developed over the years following these discoveries and have been supported recently by the important work of the Morales’ group at National Institute of Drug Abuse (NIDA) [8] (Figure 1).

Figure 1 is an illustration of the Brain Reward Cascade, which involves the release of serotonin at the hypothalamus, which stimulates enkephalin. The enkephalin then inhibits gammaaminobutyric acid (GABA) at the substantia nigra, which, in turn, regulates the amount of dopamine released at the NAc (or “reward site”). The dopamine originates in the VTA. Various receptors (including 5HT2a receptors, μ-opiate receptors, GABAA receptors, GABAB receptors, and dopamine receptors) are utilized in the reward cascade. Recent evidence demonstrates the role of the dorsal raphe nuclei in this cascade [8]. It is well-known that, under normal conditions, dopamine in the nucleus accumbens through a number of cascading events and neurotransmitter interaction works to maintain a person’s normal drives [1,3,9–13] with permission [1].

### Gentile pro-dopamine therapy with a glutaminergic-dopaminergic optimization complex (GDOC) required for long-term dopamine homeostasis

Most recent work on heroin addicts during opioid maintenance treatment [14] and alcohol abstinence [15] as well as earlier work from China [16–18] characterize via neuroimaging the mechanisms involved during these states in terms of brain reward functionality.

A feeling of well-being may be achieved only when dopamine is released at balanced “dopamine homeostatic” levels. Genetic and epigenetic abnormalities produce a dysfunction of dopamine called “dopamine resistance,” and as such, could cause aberrant cravings, even if we have not yet determined other potential opioid non-dopamine reward mechanisms as proposed by Fields’ group [19]. Consequently, there is a necessity for a compound that can target and achieve dopamine regulation (i.e., dopamine homeostasis). There is further need for a compound that can be administered to normalize brain impairments by activating the release of optimal amounts of brain dopamine at the reward site and thus, reduce excessive craving behaviors.

It is accepted that drug addiction is categorized by extensive irregularities in brain activity and neurochemistry, incorporating drug-related results on concentrations of the excitatory and inhibitory neurotransmitters glutamate and gamma-Aminobutyric acid (GABA), respectively. In healthy persons, these neurotransmitters motivate the resting state, a default state of brain activity that is also interrupted in addiction. We are in agreement with the concept that resting state functional connectivity may have clinical relevance crucial to the development of and risk for all RDS behaviors. Studies have shown that addicted individuals tend to show decreases in the glutaminergic system compared to healthy controls [20,21]. Moreover, select corticolimbic brain regions showing glutamatergic and/or GABAergic abnormalities have been similarly implicated in restingstate functional connectivity deficits in drug addiction [21]. There are many studies showing impairments of resting state functional connectivity with alcohol, opiates, cannabis, psychostimulants, nicotine, glucose, and even some behavioral addictions, further suggesting the need to find compounds that will restore normal resting state functional connectivity [22–36].

Along these lines, it has been revealed that N-Acetyl-Cysteine (NAC), compared to placebo in smokers who maintained abstinence, reported fewer cravings and higher positive effects, and concurrently presented stronger resting-state functional connectivity (rsFC) between ventral striatal nodes, medial prefrontal cortex and precuneus-key default mode network nodes, and the cerebellum [22]. Most recently, our laboratory proposed the combination of NAC with a well-known enkephalinase inhibitor and other prodopaminergic substances to combat aberrant RDS behaviors [1]. Additionally, our laboratory [23] showed that a pro-dopamine complex mixture called KB220Z induced an increase in blood-oxygen-level dependent (BOLD) activation in caudate-accumbens-dopaminergic pathways of abstinent heroin addicts, when compared to placebo1-hour after acute administration. In these abstinent heroin addicts, resting-state activity was also reduced in the putamen by KB220Z. In the second phase of this pilot study, three brain regions of interest were observed as significantly activated above resting-state by KB220Z compared to the placebo in all ten abstinent heroindependent subjects (with protracted abstinence on average of 16.9 months). Specifically, increased functional connectivity was seen in a recognized system including the dorsal anterior cingulate, medial frontal gyrus, nucleus accumbens, posterior cingulate, occipital cortical regions, and cerebellum. These results and other quantitative electroencephalography (qEEG) study outcomes propose a putative anti-craving/anti-relapse function of KB220Z in addiction by direct or indirect dopaminergic communication [37–39].

Regarding the support for the concept of long-term activation, instead of blocking dopamine release in the NAc and other relevant brain regions like the cingulate gyrus (relapse region), Willuhn *et al.* [40] pointed out that cocaine consumption, and even non-substance-associated addictive behavior, increases as dopaminergic activity declines. Habitual cocaine exposure has been linked to be reduction in D2/D3 receptors and is also linked to decreased activation in response to cues in the occipital cortex and cerebellum as indicated in a recent positron emission tomography (PET) study by Tomasi *et al.* [41]. Volkow *et al.* [42] also showed that stimulant-induced dopamine increases are markedly blunted in active cocaine abusers, despite methylphenidate-induced changes in the ventral striatum, which were associated with intense drug craving. It is our opinion that this seemingly paradoxical response is consistent with super sensitivity, as proposed earlier with the possibility of relapse, especially in *DRD2 A1* carriers [43]. In clear support of the potential for utilizing compounds that induce dopamine homeostasis in the long-term, Badgaiyan and associates [44] recently reported that at rest, the ligand binding potential (BP) was considerably increased in the right caudate of attention-deficit/hyperactivity disorder (ADHD) subjects, proposing decreased tonic dopamine release. During task performance, significantly lower ligand BP was observed in the same area, indicating increased phasic release. In ADHD, the tonic release of dopamine is attenuated, and the phasic release is enhanced in the right caudate. This characterization of the nature of deregulated dopamine neurotransmission in ADHD helps to explain earlier mixed findings of reduced or increased dopaminergic activity, which may also be the case in other RDS behaviors, including risk for opiates/opioids. Certainly, it is known that carriers of the *DRD2 A1* allele have a higher chance of relapse as reported by Dahlgren *et al.* [45]. Therefore, while we agree with the short-term utilization of United State Food and Drug Administration (FDA) medication-assisted treatments (MATs) to block excessive dopamine release leading to psychological extinction, we must also consider long-term treatment strategies such as the utilization of powerful D2 agonists like bromocriptine, which will ultimately reduce dopamine D2 expression [46]. As such, long-term, even life-long treatment with gentle pro-dopamine therapy, not potent D2 agonists, may provide dopamine homeostsis. We are therefore proposing that as an anti-opiate restoration strategy, potentially preserving dopamine activity, may be a unique and effective method of relapse prevention in opiate/opioid abuse, acute abstinence, and behavioral addictions, and warrant considerably more research.

Our essential tenet is that addiction has a high genetic inheritability factor based upon reward deficiency, a hypodopaminergic trait characteristic, and possibly even a “hyperdopaminergic” state characteristic during abstinence [15], and does not follow Mendelian inheritance (sui generis). We deem that in order to alter the continued abuse of opiates/opioids by a very significant number of people in the USA and across the world, the use of our proposed glutaminergic-dopaminergic optimization complex (GDOC), if adopted after careful consideration and well-designed research, should result in a better clinical outcome in the long-term.

As such, a new KB220 variant that can induce “dopamine homeostasis” called *GDOC*, is just one part of many other holistic approaches to treat RDS, especially during recovery. With that stated, we are aware of the various pitfalls related to all addictive behaviors, drug and non-drug related, and especially in regards to opiate addiction, there remains a large health concern with few treatment options permitted by the FDA and presently accessible. We have made important growth in our present comprehension of several features of RDS and associated addictive behaviors including neurobiology, candidate reward and additional genes, and numerous genomic-based human and animal experiments. It is our opinion, that with the advent of neuroimaging tools, the comprehension of each psychiatric disorder has risen, and we have obtained vast knowledge of brain activity and behavioral functions. Surely, Genome-wide association studies have recognized unique aims, but may indeed find real answers by gene convergence linked to top candidate genes in the final analysis. Failures of Genome-wide studies to date may be due to poor controls, whereby these so-called controls actually have hidden or unscreened RDS behaviors. Perplexity in the literature has transpired because we have not accepted the right phenotype to assess, and we have not obtained disease-free controls in several of our genetically based studies - something that is continuously ignored by our greatest minds.

Moreover, others have used modern optogenetics: opsin microbial engineering and molecular genetics models for cell-type targeting and optical strategies for guiding light through brain tissue, allowing for optical control of defined cells in living systems. Deisseroth’s group [47] recently used target transcranialmagnetic stimulation (rTMS) in a clinical study to help patients addicted to cocaine. In essence, they found that 69% of the rTMS-treated group of 32 cocaine dependent individuals compared with 19% of the control group, remained drug-free during the initial treatment phase (as tracked by urine drug tests). The rTMS treated group also reported significantly less cocaine craving. Others have proposed the utilization of transcranial magnetic stimulation (TMS) in refractory heroin addicts, especially by targeting the cingulate gyrus and NAc brain regions [48].

Our model proposes that we should begin to employ genetic testing to determine risk stratification, drug urine screening for patients in both in-patient and out-patient programs, and provide, especially during treatment and aftercare, methodology that will promote long-term “dopamine homeostasis.” In an attempt to determine the effects of our new variant of KB220z on a highly functional professional subject with a number of work-related issues, the following case reveals that chronic use of KB220z in a liquid nano form induced a number of positive effects.

The KB220z product has been developed as an aqua-nano-sized product that is non-trans-fat, having a 10–20 nano particle size. Specifically, the patented product is composed of the following ingredients in confirmed, evidence-based consumption levels: Thiamine 15 mg (1033% of Daily Value); Vitamin B6 10 mg (500%); Chromium polynicotinate (as ChromeMate^®^) 200 mcg (166%); a fixed dose mixture of amino acids and herbs called Synaptose^™^, which contains DL-Phenylalanine, L-Tyrosine, Passion Flower Extract; a Metalloglycoside^™^ Complex comprised of Arabinogalactans, N-Acetylglucosamine, Astragalus, Aloe Vera, Frankincense Resin, White Pine Bark Extract, and Spirulina; Rhodiola (as RhodiGen^™^); L-Glutamine; 5-Hydroxytryptophan (5-HTP); Thiamine Hydrochloride; Pyroxidal-5-phosphate; and Pyridoxine HCl. The corresponding placebo powder is manufactured by Cephram, Inc. (New Jersey).

## Presentation of Case

The male subject LK is 69 years of age, weighing 195 pounds with a height of 5 feet 9 inches. The subject reported taking KB220Z at a consistent dosage of one-half (0.5) ounce twice a day, starting on February 24, 2016. The self-report by his Physician was obtained after a one-month period of usage, which will be described in Box 1.

Box 1 Before taking the product, the subject was exhausted and somewhat situationally depressed. In addition to long hours of work (18 hour days), and the task of organizing many moving parts to his schedule such as working in six different locations and performing very diverse work (e.g. teaches 5 yoga classes per week, teaches psychology at the college level, runs a startup business, consults as a psychologist at a French school, runs 3 weekly groups for New Pathways Counseling Services, and supervises the clinical staff in two locations). The subject generally goes to bed at 1:30 am and gets up at 7:00 am. He also visits his mother in a nursing home twice a week. She has Alzheimer’s disease. Certainly, this intense work schedule is extremely emotionally draining for this highly functional subject.

The following, as characterized by the subject, is presented: First three to four days, I experienced sense of activation in the brain that felt like white noise approximately 30 minutes after taking it and this sensation was strong for about 45 minutes and then dissipated. This was not an unpleasant sensation. My eyesight improved slightly, like the feeling you get when you are prescribed a new pair of glasses. It is noteworthy, that I have been unable to smell anything, even strong odors like ammonia or bleach, for at least 8 years, and now I am getting glimmers of partial sent. My tinnitus has returned. Although this had been a condition that was frustrating several years ago when it started, I had gotten used to it and it had disappeared over time. With your product, it has returned, but again, it is not necessarily unpleasant this time around. Occasionally, I fall asleep watching the news in the late evening and usually I wake up and go to bed when my wife comes home at around 12:30 am. Now, when I wake up, my body and mind feel very heavy and dense. I have a similar feeling in the AM when I wake, but not as pronounced, yet, within a few seconds I am clear headed. Surprisingly, two nights in the last week, I hardly slept the entire night, but still got up clear headed. It was not an unpleasant experience perhaps because I do lots of meditation and I feel relaxed when this kind of situation occurs. So it was like I was just meditating all night. Generally, my entire mood has improved.

Moreover, I usually go to the edge of negative feelings, I notice the edge, but no longer fall over. My energy levels have improved a bit. I am more motivated to work harder in getting things done. My concentration has improved and I am clearer about the scope and purpose of each task. My ability to multi-task has improved. Socially, I am funnier, according to my wife, and more relaxed. I talk incessantly and I have had to make an effort to curb this in order to let others have their turn. I am freer at saying what I want and have had to watch more carefully what I say to patients. So again, it has caused me to have more driven speech and a bit less cautiousness.

My Clinical Interpretation is that:

1. My neurological system is working better overall (e.g. improved sensory input and overall activation of the brain).
2. My sleep cycle has improved, perhaps with an increase and/or more efficient rapid eye movement (REM) (alpha) segment of the cycle (e.g. heaviness upon waking).
3. An increase in my Behavior Activating System (+dopamine/+reward), while I have a decrease in the Behavior Inhibition System (+serotonin/−caution).

The interpretation of the subject favors a positive response over a 30-day period and is highlighted by previous research suggesting neurochemical-induced benefits from KB220 variants.

## Discussion

Albeit this is limited to only one case, the effects self-reported by the subject and his subsequent interpretation, is backed by many studies on KB220 in various forms [49]. In fact, the overall activation of brain reward circuits has been shown in previous studies including both human [50] and animal models [51]. Specifically, as specified earlier in this report, KB220Z variant induced an increase in BOLD activation in caudate-accumbens-dopaminergic pathways of abstinent heroin addicts when compared to placebo one-hour after acute administration. In these abstinent heroin addicts, resting-state activity was also reduced in the putamen by KB220Z. In addition, three brain regions of interest were observed as significantly activated above resting-state by KB220Z compared to the placebo in all ten abstinent heroin-dependent subjects (with protracted abstinence on average of 16.9 months). Furthermore, increased functional connectivity was seen in a putative system comprised of the dorsal anterior cingulate, medial frontal gyrus, nucleus accumbens, posterior cingulate, occipital cortical regions, and cerebellum.

In terms of positive sleep aspects, earlier studies have shown that KB220Z can actually improve sleep [52] and even eliminate posttraumatic stress disorder (PTSD)/ADHD induced unwanted terrifying lucid dreams [53–54]. Along these lines, qEEG studies utilizing KB220Z has revealed reduced widespread theta activity with concomitant increase in both alpha and low beta waves in abstinent psychostimulant abusers [37] as well as in alcoholics and heroin addicts [38]. This general effect has been linked to a calming action that can occur following at least 10–20 neuro feedback sessions [37]. The subject described his sleep benefit as being similar to meditation. This is underscored by the recent work showing that Yoga meditation causes a 65% increase in dopamine release [55].

The self-interpretation by the subject can be further explained by neuroimaging results by others for both alcohol and heroin dependent subjects during drug maintenance and protracted abstinence.

Recent work from Zhang *et al.* [14] used Granger causality analysis to investigate directional causal influences among the brain circuits in heroin-dependent individuals during opioid maintenance treatment (HDIs-OMT) and non-opioid users. Their outcomes presented a diminished connectivity between the caudate nucleus involved in resolving the reward circuit and other brain areas and also a diminished connectivity between the anterior cingulate cortex and medial prefrontal cortex involved in resolving inhibitory control. In contrast, HDIs-OMT presented greater effective connectivity between the hippocampus and amygdala involved in mediating learning-memory, and the anterior cingulate cortex associated with mediating inhibitory control while the putamen mediated learned behaviors, proposing that the hippocampus and amygdala may drive the memory circuit to supersede the regulator circuit and push the learned behavior in HDIs-OMT. These interesting findings may provide insight into treatment targets. The authors correctly suggest that continued neural effects of opioid addiction on methadone maintenance including hyper activation in the memory circuit and damage in the control circuit help the role of the memory circuitry in relapse and may redefine targets for treatment. Interestingly, our findings with KB220Z, showing an enhanced resting state in abstinent heroin addicts accompanied with an enhanced functionality in the control circuit (cingulate gyrus) as well as a reduced or balanced activity of the hippocampus putamen, seems to help explain the delayed onset of relapse in poly-drug abusers obtained in earlier work [50].

In alcohol dependent and abstinent subjects, and in rodent models, surprisingly Hirth *et al.* [15] found convergent evidence revealing a “hyper-dopaminergic” state during a three-week long alcohol abstinence in rats that seems to agree with their post-mortem human data. While this could be the actual fact, it would be of interest to apply the Granger causality analysis as described by Zhang *et al.* [14] to provide for a clearer view as to exactly which regions of the brain maybe “hypodopaminergic” compared to “hyper-dopaminergic,” as reported for maintained heroin addicts as well as abstinent heroin addicts revealed in earlier studies by the same group in China [16–18]. Moreover, it would have been important to characterize the alcoholic cohort presented by the Hirth *et al.* [15] study by genotyping the entire sample and then by genotype in order to re-evaluate the results to eliminate DNA polymorphic traits. However, even until this question is resolved, we believe that our best approach for targeting relapse prevention in at least opiate/opioid dependence during recovery is to balance endorphinergic-glutaminergic-dopaminergic brain function by coupling D-Phenyalalnine and NAC as novel therapeutic modalities as found in KB220Z [1].

## GDOC and Blood Brain Barrier

The passage of molecules in and out of the central nervous system (CNS) is highly regulated by the blood brain barrier (BBB) [56,57]. The brain microvasculature is comprised of three layers: endothelial cells, astrocytes end-feet, and pericytes [56]. The BBB is a single layer of specialized endothelial cells located within the capillaries that deliver blood to the brain; they are referred to as brain capillary endothelial cells (BCECs) [57]. BCECs are connected by highly resistant junctions, which are polarized into luminal (blood–facing) and abluminal (brain–facing) plasma membrane domains [58]. The selective permeability of the BCECs protects the brain by limiting the passage of harmful molecules into the CNS [59].

The BBB is the reason for the instruction to take the first dose of oral GDOC before breakfast and the second dose before dinner after at least a two-hour protein fast. It is well established that an aminoacid carrier system in the brain means that amino-acids derived from protein compete for a place on the carrier to enter the brain from the periphery. Since GDOC contains select precursor amino acids to build neurotransmitters, taking the oral GDOC without food present allows for the highest possible amounts of these select amino-acids to enter the brain and start the synthesis of specific neurotransmitters like serotonin, GABA, and Dopamine.

Neurotransmitter transport across the blood brain barrier is mediated by known and specific transporters. The transporters are specific for various neurotransmitters allowing access from the brain to the blood and vice versa [60]. It is important to note that the aminoacids in this complex are in the lipophilic form, which enables these neurotransmitter precursors to enter the blood brain barrier [BBB]. There is also evidence that Chromium salts as well as Rhodiola also enters the brain as well [61,62]. More importantly, it is well-known that stress increases the permeability of the BBB [63].

## Conclusion

These results and other qEEG and neuroimaging studies reveal an improved mood, work-related focus, and sleep. These effects, as indicated by the subject as a subjective feeling of brain activation, maybe due to direct or indirect dopaminergic interaction. We must await more research in a larger randomized placebo-controlled study to actually map the role of KB220Z, especially in a nano-sized product to determine the possible effects on circuit inhibitory control and memory banks, and the induction of dopamine homeostasis independent of either hypo- or hyper-dopaminergic traits/states.
